# Experience Curve With the Cone Procedure for Ebstein’s Anomaly: Effect on Cost and Resource Utilization

**DOI:** 10.1016/j.jacadv.2024.101104

**Published:** 2024-07-13

**Authors:** Peter Chiu, Abdelilah El Azfi, Brandon Kwon, Sitaram M. Emani, Gerald Marx, Pedro J. del Nido

**Affiliations:** a Department of Cardiac Surgery, Boston Children’s Hospital, Boston, Massachusetts, USA; b Department of Cardiology, Boston Children’s Hospital, Boston, Massachusetts, USA

**Keywords:** experience curve, health economics, public policy, regionalization, specialty care

## Abstract

**Background:**

The volume-outcome relationship is well-known in health care. The Experience Curve, initially developed by the Boston Consulting Group for manufacturing, offers insight on this relationship and has never before been applied to health care.

**Objectives:**

The purpose of this study was to determine the effect of experience on cost and resource utilization for the Cone procedure.

**Methods:**

We performed a retrospective review of patients who underwent Cone reconstruction for Ebstein’s anomaly at Boston Children’s Hospital between October 2010 and October 2021. Cardiopulmonary bypass time and aortic cross clamp time over time were evaluated using exponential regression to assess the surgeon-level learning curve. At the hospital level, length of stay and cost over time were assessed using exponential regression.

**Results:**

There were 115 patients included in the study. Median hospital length of stay was 7.9 days (IQR: 6.4-10.2 days) with a decline of 3.3% per year. Median intensive care unit [ICU] length of stay was 3.2 days (IQR: 2-5.7 days) with a decline of 10.5% per year. Adjusted direct costs indexed to 2020 prices fell by 4% per year. There was no statistically significant change in cardiopulmonary bypass or aortic cross clamp time. In mediation analysis, the reduction in cost was completely accounted for in the decline in ICU length of stay. ICU length of stay was correlated with duration of intubation.

**Conclusions:**

Increasing familiarity with Ebstein’s anomaly and Cone reconstruction led to a reduction in resource utilization.

The strong relationship between procedural volume and outcome has been demonstrated in many facets of cardiac surgery including aortic surgery, coronary artery bypass grafting, and congenital heart surgery.^1^, ^2^, ^3^, ^4^, ^5^ The primary outcome of interest in these studies has been mortality. However, mortality may not be precise enough of a measure to delineate differences between complex systems. The focus in health care economics has shifted toward quality of care and value-based delivery of health care. Goals of such an approach are to minimize costs while maximizing outcome increasing the sustainability of the health care system.

In this regard, focusing the provision of highly specialized care at a limited number of centers of excellence has the opportunity to achieve such a task. Increasing experience has been associated with a higher rate of mitral valve repair in addition to lower risk for reoperation in a population-level study of New York State.^4^ The goal of reducing cost is a distinct phenomenon. The Boston Consulting Group introduced the concept of the “Experience Curve” suggesting that the cost of a repetitive task decreases by a fixed percentage with the doubling of the accumulated volume of production.^6^ The intrinsic slope may vary across manufacturing industries; however, the overall relationship holds true. The delivery of health care differs fundamentally from manufacturing given the complex systems involved. Despite this, the establishment of Centers of Excellence would suggest that there is a role of increasing experience with creating high-reliability systems able to provide high-quality at low cost.

To test the hypothesis that the experience curve may be replicated in health care, we sought to identify a procedure of high complexity with respect to both surgical technique and postoperative management. Ebstein’s anomaly is a rare lesion in which the tricuspid valve and right ventricle are severely abnormal leading to tricuspid regurgitation and right ventricular dysfunction. Every effort is taken to repair these valves rather than replace them due to the risks associated with prosthetic valve replacement. Biologic prostheses have limited durability, and mechanical prostheses require anticoagulation with attendant risks for hemorrhage and thrombosis. Moreover, the availability of smaller-sized prostheses for children is limited, and these devices do not allow for growth. The Cone procedure was first reported by da Silva et al and has proven to be the single best technique for tricuspid valve reconstruction in Ebstein’s anomaly.^7^, ^8^, ^9^ Postoperatively, these patients may be critically ill with severe right ventricular dysfunction requiring advanced and high-cost therapies such as inhaled nitric oxide. We undertook the current study to determine the effect of experience on cost and resource utilization for the Cone procedure at our quaternary referral center.

## Methods

After approval from the Boston Children’s Hospital Institutional Review Board (IRB-P00043360, Approval 8/2022), a query was performed of our institutional cardiac surgery database, and a retrospective review was performed for patients who underwent Cone reconstruction for Ebstein’s anomaly of the tricuspid valve at Boston Children’s Hospital between October 2010 and October 2021. Patients who underwent concomitant operations other than atrial septal defect closure, arrhythmia ablation, bidirectional Glenn, or ventricular septal defect closure were excluded from the study to create a homogeneous cohort.

Ebstein’s anomaly is an archetype for specialized care due to the overall rarity of the lesion, and the unique challenges that it presents both the surgeon and the institution. The Cone procedure is a very unique operation with high technical complexity in part related to the variability of the lesion. Moreover, inherent to the disease process is an increased risk for isolated right ventricular failure, which presents distinct management challenges. Patients often require fastidious management of volume status in addition to administration of inhaled nitric oxide, optimization of ventilator settings, and use of inotropic medications.

### Outcomes

The parallel contributions of surgeon-level and hospital-level effects have been explored by Birkmeyer et al suggesting that these 2 elements independently affect postoperative survival.^1^^,^^2^ To assess surgeon-level effects and the effect of individual surgeon learning curve, we used changes in cardiopulmonary bypass time and aortic cross clamp time over time. Decreased operative time has been associated with increased efficiency and experience.^10^ Perioperative mortality was evaluated as a secondary outcome. However, given the low incidence of mortality in the cohort, we used incidence of moderate tricuspid regurgitation on discharge echocardiogram as an additional means to assess surgeon-level effects.

For hospital-level effects, we examined total postoperative hospital length of stay, postoperative intensive care unit (ICU) length of stay, and costs. Costs were assigned as direct costs, which amounted to controllable patient care expenses, or indirect costs, which were uncontrollable expenses including depreciation of capital assets, marketing costs, etc. Costs were adjusted to 2020 prices using consumer price index methodology for health care reported by the United States Bureau of Labor Statistics.^11^ In brief, the consumer price index accounts for the total reimbursement (medical care services and medical care commodities) tracking inflation. This allows for accurate comparisons from year to year. Cost outliers were identified as patients in the 90th percentile of the entire experience.

### Statistical analysis

For surgeon-level effects, duration of cardiopulmonary bypass and aortic cross clamp time were assessed using a mixed effects model with surgeon as a random effect in order to account for individual surgeon-level variation. Additionally, mixed effects logistic regression was then used to evaluate the change in moderate or greater tricuspid regurgitation at discharge over time, once again accounting for individual surgeon-level variation using surgeon as a random intercept.

Given the exponential relationship suggested by prior investigations into the Experience Curve, an exponential model was constructed in order to evaluate hospital-level effects of increasing experience on total postoperative hospital length of stay, postoperative ICU length of stay, and direct costs. Additionally, the proportion of cases per year that was greater than the mean for the period under study was plotted over time and this was evaluated with the Mann-Kendall test to identify the presence of a monotonic secular trend.

As a sensitivity analysis, 4 operations that have had fairly standardized approaches for a prolonged period of time (the Fontan procedure, repair of Tetralogy of Fallot, complete atrioventricular canal defect, and the Norwood procedure) were evaluated for the presence of a similar exponential decline in hospital length of stay and ICU length of stay during the same time period. As an additional sensitivity analysis, the 90th percentile, that is cost outliers, for patients undergoing surgery were removed from the data set, and the analysis was repeated in order to assess the effect of outliers on the trend. Finally, a third sensitivity analysis was performed restricting the population of patients to those who underwent surgery prior to the establishment of the Enhanced Recovery After Surgery (ERAS) program in 2018.

Given the potential relationship between ICU length of stay and cost, mediation analysis was then performed. The natural log of cost was regressed on time in years, as above. The natural log of ICU length of stay was also regressed on time in years, as above. Cost was then regressed on the sum of time in years and the log of ICU length of stay. From this, the average causal mediation effect was estimated. A sensitivity analysis eliminating the 90th percentile of patients was then performed to evaluate the effect of cost outliers on the mediation analysis. In an effort to elucidate mechanisms for change in postoperative ICU length of stay, postoperative ICU length of stay was regressed on number of days intubated in order to explore the potential relationship.

Absolute counts are reported with percentages. Continuous variables are reported with median (IQR). A 2-tailed *P* value <0.05 was considered statistically significant. Due to the exploratory nature of the analysis, no correction was made for multiple testing.^12^ All analyses were performed using R-4.2.1 (R Foundation).

## Results

There were 115 patients who were included in the study. The median age was 11 years (IQR: 6-16 years), and 64 patients (55.7%) were male. A single surgeon performed 100 of the 115 operations (87%). Preoperative tricuspid regurgitation was greater than moderate in 77 (67%) and was mild or less in 9 (7.8%), Table 1.

**Table 1:** Demographic Variables (N = 115)

Male sex	64 (55.7%)
Age, y	11.00 (6.00-16.00)
Weight, kg	35.60 (20.45-60.20)
Height, cm	142 (119-166)
Reoperation	1 (0.9)
Preoperative grade of tricuspid regurgitation	
0+	1 (0.9%)
1+	8 (7.0%)
2+	29 (25.2%)
3+	13 (11.3%)
4+	64 (55.7%)

Values are n (%) or median (IQR).

### Surgeon-level effects

There were a total of 4 surgeons who performed the Cone procedure during the period under study. The median number of cases per year during the study period was 8.5 (IQR: 7-12.25) (Supplemental Figure 1). Median cardiopulmonary bypass time was 153 minutes (IQR: 130.5-176 minutes), and median aortic cross clamp time was 84 minutes (IQR: 69.5-106.5 minutes). There was no change in cardiopulmonary bypass time or aortic cross clamp time over the study period using linear or exponential regression with and without surgeon as a random effect (Supplemental Figures 2 and 3). Moderate or greater tricuspid regurgitation was observed at discharge in 12.2% of patients (14/115) (Supplemental Figure 4). There was no change over time by logistic regression with and without surgeon as a random effect.

There were 7 reoperations during the same admission for recurrence of tricuspid regurgitation. Additionally, 2 patients required return to the operating room for closure of an atrial level communication. One patient required reoperation for bidirectional Glenn in the setting of right ventricular dysfunction, and one patient required reoperation for right coronary occlusion also in the setting of right ventricular dysfunction. Three patients (2.6%) required postoperative pacemaker for complete heart block (Table 2). In follow-up, 5 patients required reoperation on the tricuspid valve at a mean of 3.6 years following the index operation with one requiring eventual replacement. There has been one death in follow-up.

**Table 2:** Outcome Variables (N = 115)

Adjusted total costs, 2020 dollars	$70,705 ($56,599-$91,395.50)
Adjusted direct costs, 2020 dollars	$43,348 ($33,018.50-$58,306.50)
Cardiopulmonary bypass time, min	153 (130.5-176)
Fibrillation time, min	39.50 (23.75-58.25)
Aortic cross clamp time, min	84.0 (69.5-106.5)
Postoperative hospital length of stay, d	7.90 (6.39-10.18)
Postoperative ICU length of stay, d	3.16 (2.00-5.69)
Tricuspid regurgitation on discharge echocardiogram	
0+	40 (34.8%)
1+	61 (53.0%)
2+	14 (12.2%)
Mediastinitis	0 (0%)
Postoperative permanent pacemaker	3 (2.6%)
Re-exploration for bleeding	0 (0%)
Postoperative mechanical circulatory support	2 (1.7%)

Values are median (IQR) or n (%).

ICU = intensive care unit.

### Hospital-level effects

**Figure 1:**
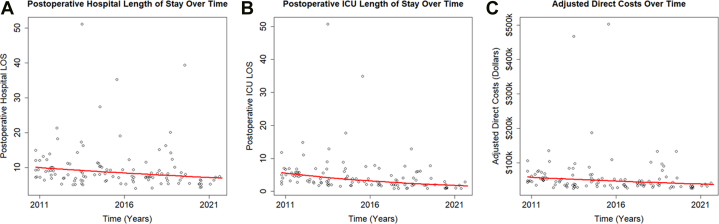
Plot of Resource Utilization and Cost Over Time (A) postoperative hospital length of stay (LOS) declined 3.3% per year [main figure is magnified to demonstrate exponential relationship; inset, overall postoperative hospital LOS for the cone procedure over time]; (B) postoperative intensive care unit (ICU) LOS declined 10.5% per year [main figure is magnified to demonstrate exponential relationship; inset, overall postoperative ICU LOS for the cone procedure over time]; (C) adjusted direct cost indexed to 2020 price declined 4% per year [main figure is magnified to demonstrate exponential relationship; inset, overall relationship adjusted direct cost for the cone procedure over time].

Median hospital length of stay was 7.9 days (IQR: 6.4-10.2 days) with a decline of 3.3% (95% CI: 0.7%-5.8%) per year using exponential regression (Figure 1A). Median ICU length of stay was 3.2 days (IQR: 2-5.7 days) with a decline of 10.5% (95% CI: 6.9%-14.0%) per year (Figure 1B). Finally, median adjusted direct cost was $43,348 (IQR: $33,018.50-$58,306.50) indexed to 2020 prices, and there was a 4% (95% CI: 1.1%-6.9%) decline per year (Figure 1C). Absolute case number was not a significant predictor of either cost or length of stay in exponential regression. Age, sex, weight, and degree of preoperative tricuspid regurgitation did not reach statistical significance in a multivariable regression model and so were excluded.

Our sensitivity analysis demonstrated no evidence of an overall trend in declining hospital length of stay or ICU length of stay for the Fontan procedure, repair of Tetralogy of Fallot, repair of complete atrioventricular canal, or the Norwood procedure (Supplemental Figures 5 to 8). The length of stay increased slightly for the Norwood procedure over the study period. Additionally, after limiting the analysis to patients prior to the institution of an ERAS program (n = 84), hospital length of stay no longer demonstrated a statistically significant decline over time, *P* = 0.08. However, the observed decline in ICU length of stay remained significant: 10.4% (95% CI: 4.2%-16.2%). The effect of experience on adjusted direct cost also remained statistically significant: 5.1% (95% CI: 0.05%-10.0%).

### Outlier care

**Figure 2:**
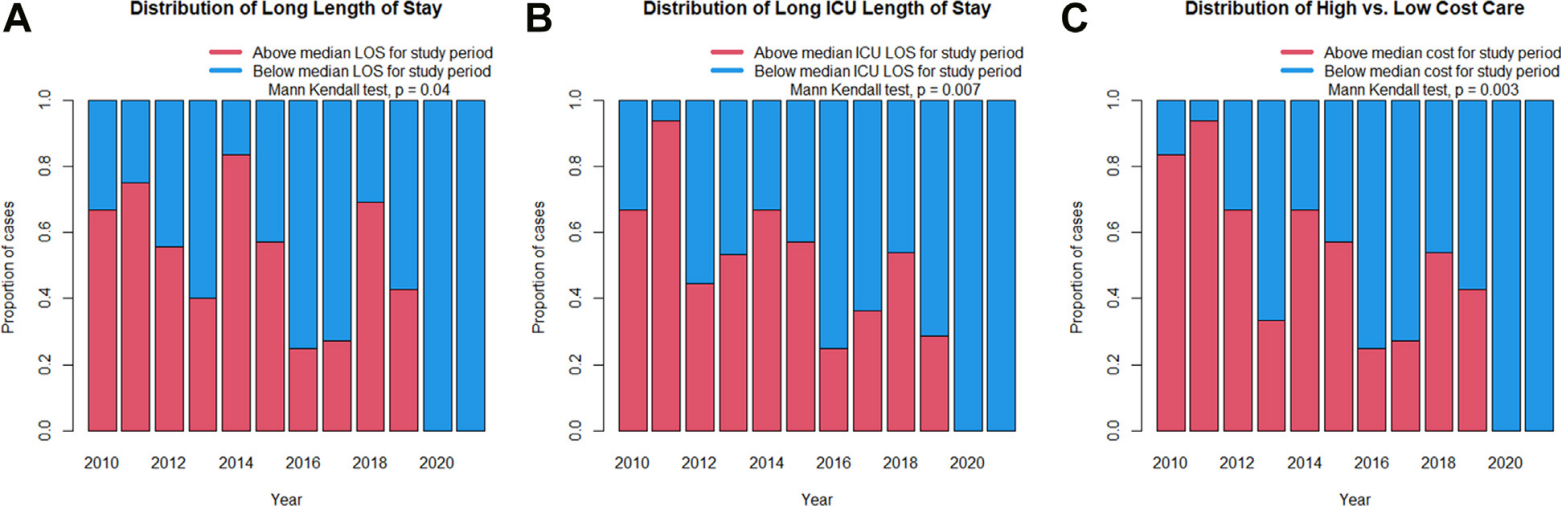
Proportion of Cases Per Year Greater Than the Median Value During the Study Period Suggesting Both a Reduction in the Overall Cost Over Time and a Reduction in the Number of Outlier Patients The decline in the number of outlier patients appears to be associated with improved quality. (A) The proportion of cases above the median hospital length of stay declined over time and was 0% in the final two years of the study (Mann-Kendall test, *P* = 0.04); (B) the same relationship held for the proportion of cases above the median ICU length of stay with an overall decline over time (Mann-Kendall test, *P* = 0.007); (C) mirroring the decline in overall cost over time, the proportion of cases above the median cost over the entire study period also declined over time (Mann-Kendall test, *P* = 0.003).

Outlier care represented a substantial burden on the system. The top 12 patients (10%) with respect to resource utilization accounted for 296.8 hospital days (26.5%) and 200.6 ICU days (36.5%) as compared to patients in the bottom 50%, who accounted for 356.8 (31.8%) and 113.7 (20.7%) hospital days and ICU days, respectively. Additionally, those 12 patients in the top 10% for adjusted direct cost represented $2,172,894 or 32.1% of the overall combined adjusted direct cost for the cohort, $6,758,892; the bottom 50% only accounted for $1,912,158 or 28.3% of the overall combined cost. The proportion of cases per year that were greater than the median total hospital length of stay (7.9 days) declined over time from a peak of 83% in 2014 to 0% in 2020 and 2021, (*P* = 0.04) (Figure 2A). A similar monotonic trend was the case for the proportion of cases per year that were greater than the median ICU length of stay for the time period under study (3.2 days) from 94% in 2011 to 0% in 2020 and 2021 (*P* = 0.007) (Figure 2B). The proportion of cases above the median adjusted direct cost ($43,348) for the time period also declined substantially from a peak of 93.8% in 2011 to 0% in 2020 and 2021 (*P* = 0.003) (Figure 2C).

Cost outliers, that is patients in the 90th percentile, had longer cardiopulmonary bypass time, 200 minutes (95% CI: 176.5-223.5 minutes) compared with 144.5 minutes (95% CI: 130.5-166.25 minutes), *P* = 0.001. These patients were also more likely to have required placement of a pacemaker (16.7% vs 1%, *P* = 0.02), mechanical circulatory support (16.7% vs 0%, *P* = 0.003), and unplanned reoperation (25% vs 3.9%, *P* = 0.02). These intermediate outcomes—pacemaker placement, mechanical circulatory support, and unplanned reoperation—were substantial potential sources of increased cost. However, a composite of these intermediate outcomes did not appear to vary over time. As a further sensitivity analysis, these cost outliers (n = 12) were removed from the data set and an exploratory analysis was further performed to assess the effect of the experience curve on the routine patients. Following removal of these patients, the exponential relationship was preserved. Hospital length of stay declined 3.5% (95% CI: 1.8%-5.2%) per year. ICU length of stay declined 10.2% (95% CI: 7.3%-12.9%) per year. Adjusted direct cost declined 4.0% (95% CI: 2.2%-5.7%) per year. This phenomenon suggests that a reduction in the number of cost outliers over time was not the primary mechanism by which the cost and length of stay were reduced (Supplemental Figure 9).

### Mediation analysis

Each additional day in the ICU was associated with a linear increase in both the adjusted direct costs ($10,041.60, *P* < 0.001) and adjusted total costs ($15,322.40, *P* < 0.001) compared with the adjusted direct cost ($2,258.60, *P* < 0.001) and adjusted total cost ($3,940.20, *P* < 0.001) associated with an additional day on the surgical ward. The average causal mediation effect, that is, the effect of time on cost that was mediated by ICU length of stay, was larger than the total effect. This would suggest that factors contributing to reduced ICU length of stay more than made up for the rise in cost seen outside of this effect. Testing for the interaction between ICU length of stay and time, the interaction was significant (*P* < 0.001), and the cost of each additional day in the ICU increased by $765.20 per year. In our sensitivity analysis eliminating cost outliers, time in years did not have a direct effect on adjusted direct costs (*P* = 0.60) suggesting that the entire cost reduction was mediated by the intensity of ICU care. Duration of postoperative ICU length of stay was highly correlated with the number of days intubated (adjusted r^2^ = 0.69 and *P* < 0.001) (Supplemental Figure 10).

## Discussion

At a single institution, increasing experience with the Cone reconstruction for Ebstein’s anomaly resulted in an exponential decline in both length of stay and cost (Central Illustration). The decline in cost appeared to be primarily mediated by a decrease in ICU length of stay over time without any meaningful surgeon-level learning curve. This finding is distinct from the existing literature on the volume-outcome relationship. Whereas higher volume centers have previously been associated with reduced mortality, length of stay, and cost, when compared to lower volume centers^1^^,^^2^^,^^13^ application of the experience curve allows for longitudinal assessment of a single center over time.

**Central Illustration:**
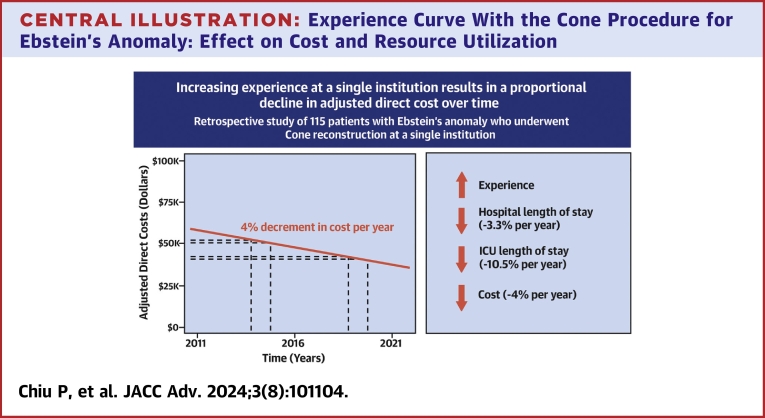
**Experience Curve With the Cone Procedure for Ebstein's Anomaly: Effect on Cost and Resource Utilization** The adjusted direct cost for the Cone procedure declined proportionally over time suggesting that increasing experience was associated with improved efficiency.

The role of experience in reducing resource utilization is likely multifactorial with both surgeon-level and hospital-level elements. At the surgeon level, Burt et al^10^ have demonstrated that with increasing experience, individual surgeon efficiency may be improved with reduced mortality, cardiopulmonary bypass time, and aortic cross clamp time as seen in a single institutional retrospective cohort study of the surgical learning curve. However, the learning curve in the study by Burt et al was a function of years since postgraduate training thus likely secondary to the early career learning curve related to gaining independence. In the current study, the surgeon-level learning curve did not appear to be a factor. This was likely related to the high level of experience of the surgeon performing the majority of the operations.

Despite Boston Children’s Hospital already being a high volume pediatric cardiac surgery center, there was a distinct decline in resource utilization as evidenced by reduced ICU length of stay and adjusted direct costs with increasing experience. The ICU is managed by a large number of cardiac intensivists, nurse practitioners, residents and fellows in training, respiratory therapists, and bedside nurses, the reduction in length of ICU stay speaks to the system-wide experience curve and the unique postoperative management considerations that appear to be separate from the typical care of infants and children after congenital heart surgery, which saw no secular trend in length of stay during our study period. ICU length of stay was highly correlated with the number of days intubated. The increasing familiarity with the lesion and the predicted postoperative course of these patients may be an explanation for the reduced duration of intubation independent of an ERAS program, which was introduced later in the study period in 2018.^14^ This phenomenon underlines the importance of specialization in the care of rare and complex lesions and may be an argument for regionalization of specialty care similar to the calls for regionalization of aortic surgery in the adult cardiac surgery literature.^15^

In an analysis of Healthcare Cost and Utilization Project data from 2011, hospital stays requiring ICU services represented 47.5% of cost but only 26.9% of hospitalizations indicating that ICU utilization represents a profound cost in the health care system.^16^ Furthermore, recognizing that longer length of stay is symptomatic of increasing ICU complexity, our mediation analysis suggested that the overall reduction in adjusted direct cost was primarily related to reduced ICU resource utilization, which includes a combination of costs related to personnel, use of support devices (including a ventilator or extracorporeal membrane oxygenation), and medications (including inhaled nitric oxide).^17^, ^18^, ^19^ Reasons for the reduction in ICU resource utilization may be related to improved surgical decision-making in the operating room, better anesthetic and analgesic management in the perioperative period, and improved recognition of potential issues in the ICU each with an overall effect of preventing ICU morbidity. Furthermore, high-volume centers with standardized protocols for patient management may develop or adapt protocols for new complex procedures compared to lower volume centers. In our sensitivity analysis, the relationship between ICU length of stay and cost was still overwhelming after eliminating cost outliers, and the overall exponential relationship predicted by the experience curve was maintained. Increasing experience appeared to result in a broad-based improvement in ICU care of these select patients.

In a study of Medicare patients undergoing coronary artery bypass grafting, the proportion of patients with outlier payments tracked well with quality, as defined by a composite of risk-adjusted mortality rate and hospital volume, with a rate of outlier payments up to 10.8% in the lowest quintile of quality as compared to 6.7% in the highest quintile of quality.^20^ In this way, the experience curve depicts the positive evolution of quality over time at our individual facility. Importantly, addressing cost outliers is a critical element in reducing overall cost. The top 10% of patients in both ICU length of stay and adjusted direct costs for our cohort accounted for more than the bottom 50% of patients in each category, respectively. However, despite the significant contribution to cost and length of stay from the outlier patients in our report, the overall exponential decline in both length of stay and cost was not primarily a function of eliminating outliers over time.

The decline in cost seen in the clinical setting differs from examples of the experience curve in manufacturing given that increasing institutional experience in the management of related relevant phenomena, for example, right ventricular dysfunction following left ventricular assist device placement, may also contribute to the overall institutional experience. This may explain the statistically significant decline over time that failed to reach statistical significance when considering the change in resource utilization per procedure performed, that is, accumulated volume of production. This suggests that comprehensive centers of excellence may have synergistic programs that allow for improved efficiency across a broad range of services with the development of breadth and depth of expertise.^15^

### Study Limitations

This is a retrospective single-center cohort study, and some limitations exist. The establishment of an ERAS program in 2018 may have contributed to a reduction in cost and length of stay, though it is important to note that much of the reduction in resource utilization was seen prior to the establishment of this program, which affected only the final 3 years of the study. Additionally, given the significant right ventricular dysfunction present postoperatively, nearly half of the patients were not extubated within the first postoperative day and thus were not eligible for our institution’s ERAS program. In our sensitivity analysis, there was no evidence of a secular trend toward improved hospital length of stay or ICU length of stay with the Fontan procedure, repair of Tetralogy of Fallot, complete atrioventricular canal, or the Norwood procedure suggesting that a global improvement in care at our facility could not be wholly responsible for the decline in resource utilization. These operations may already be near a horizontal asymptote at which only marginal gains may be realized.

## Conclusions

The decline in resource utilization over time with increasing experience in the Cone reconstruction for Ebstein’s anomaly at a single quaternary center is a novel application of the experience curve to health care. Given the rarity of certain lesions, regionalization of specialized care may accelerate the experience curve at select centers with the potential for reductions in cost and improvements in outcome. The role for increased specialization throughout the field of cardiac surgery and specifically in congenital heart surgery warrants further investigation.

Perspectives **COMPETENCY IN SYSTEMS-BASED PRACTICE:** An understanding of the relationship between cost and institutional experience may inform strategies to improve care.

**TRANSLATIONAL OUTLOOK 1:** Additional research is needed to better understand the surgeon-level and institutional-level effects on the reduction in cost.

**TRANSLATIONAL OUTLOOK 2:** The extent to which the experience curve may be applied to other procedures of varying complexity—either by virtue of technical challenges or institutional resource utilization—is not well understood.

## Funding support and author disclosures

The authors have reported that they have no relationships relevant to the contents of this paper to disclose.
