# Supplementary material for: Experience Curve With the Cone Procedure for Ebstein’s Anomaly: Effect on Cost and Resource Utilization
Source: JACC Adv. 2024 Jul 13;3(8):101104. doi: 10.1016/j.jacadv.2024.101104 (PMC11299563; doi:10.1016/j.jacadv.2024.101104)
Supplement: Supplementary Data [file mmc1.pdf]

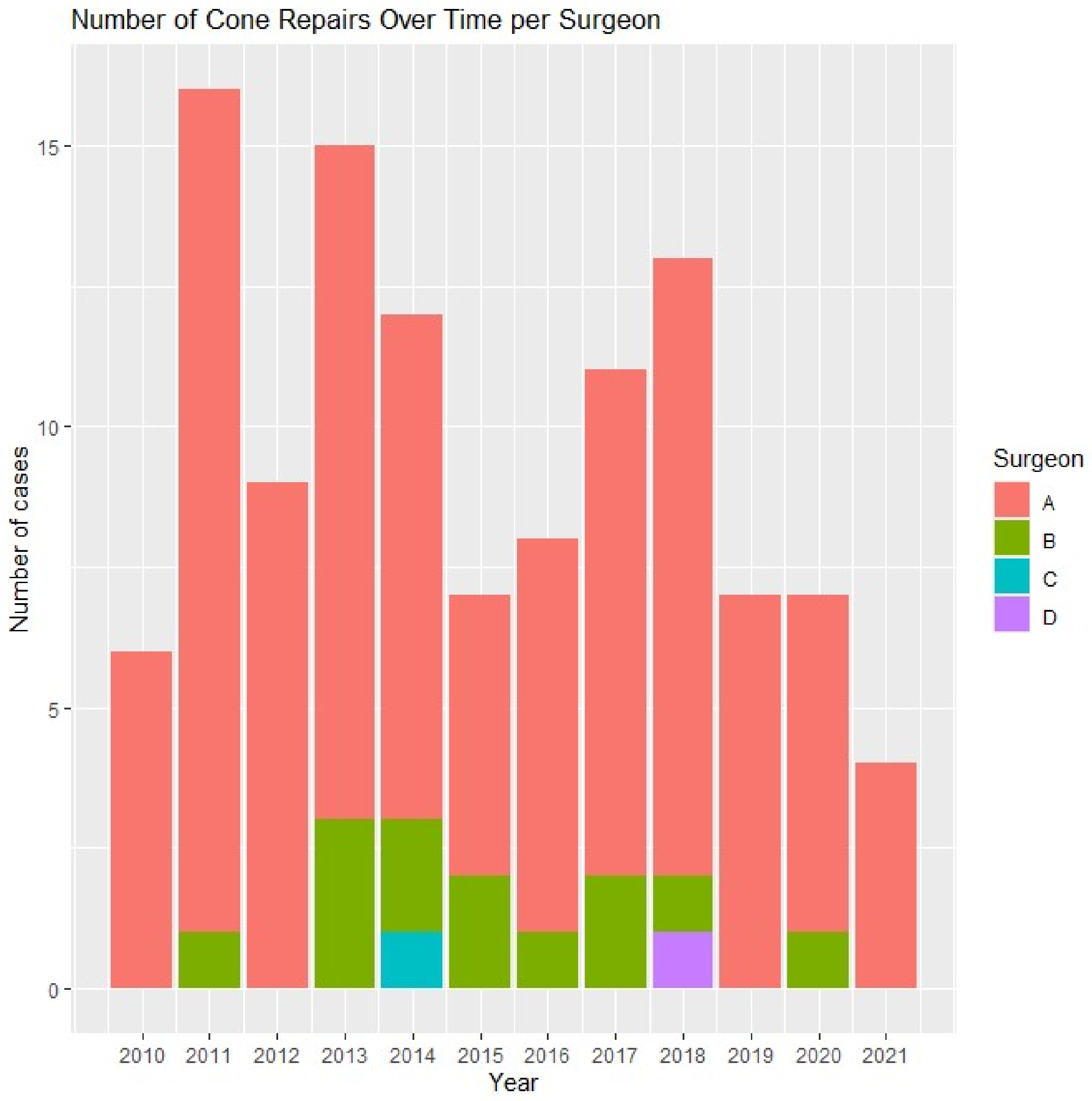

Supplemental Figure 1. There were a total of 4 surgeons who performed the cone procedure during the period under study, and 1 surgeon performed 100 of the 115 cases. The median number of cases per year during the study period was 8.5 (IQR: 7 to 12.25).

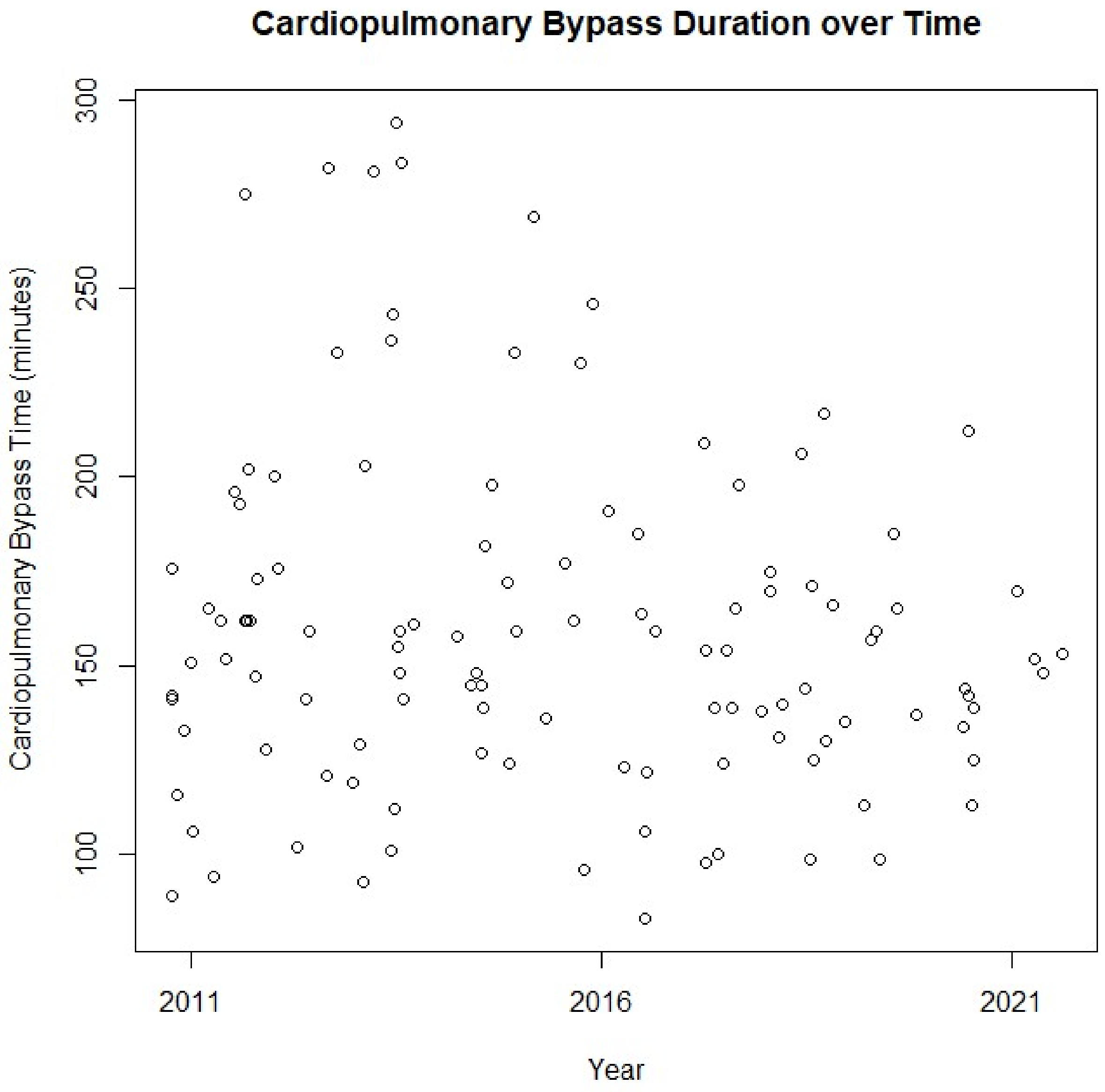

Supplemental Figure 2. There was no statistically significant change in the duration of cardiopulmonary bypass time over time.

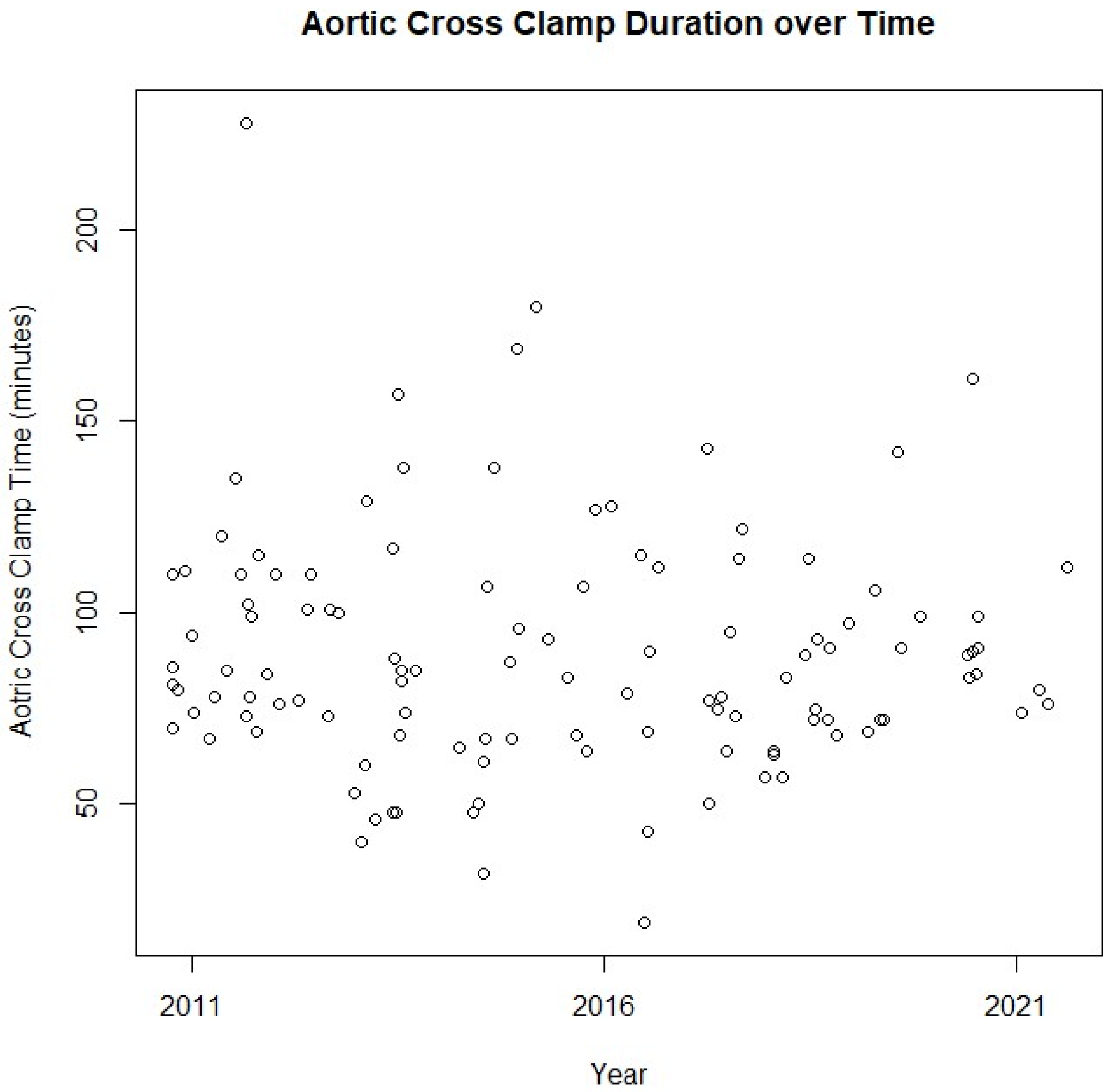

Supplemental Figure 3. There was no statistically significant change in the aortic cross clamp time over time.

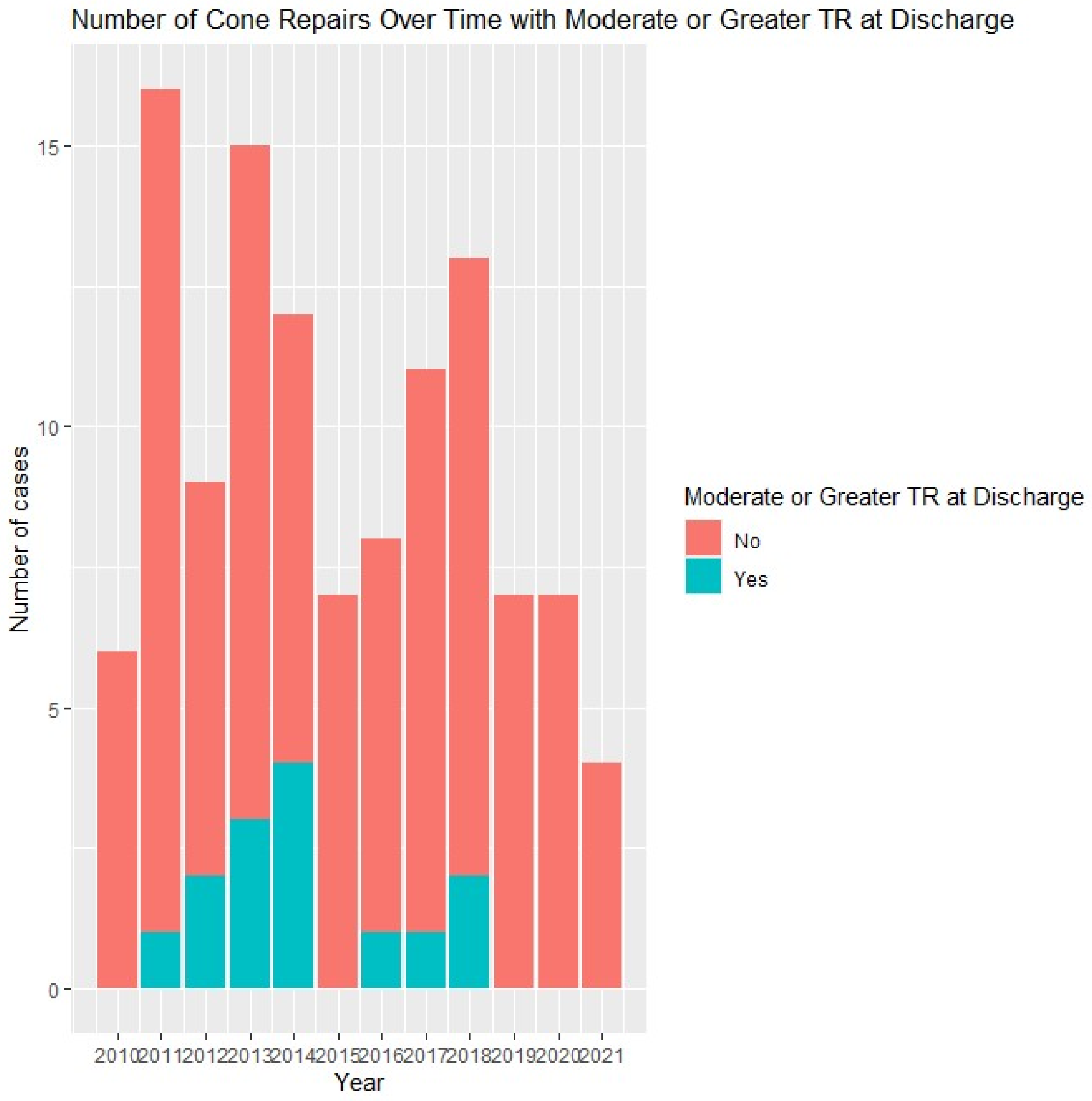

Supplemental Figure 4. Moderate or greater tricuspid regurgitation was noted in 14 patients (12.2%) at discharge. All patients were noted to have moderate and none of the patients had severe tricuspid regurgitation.

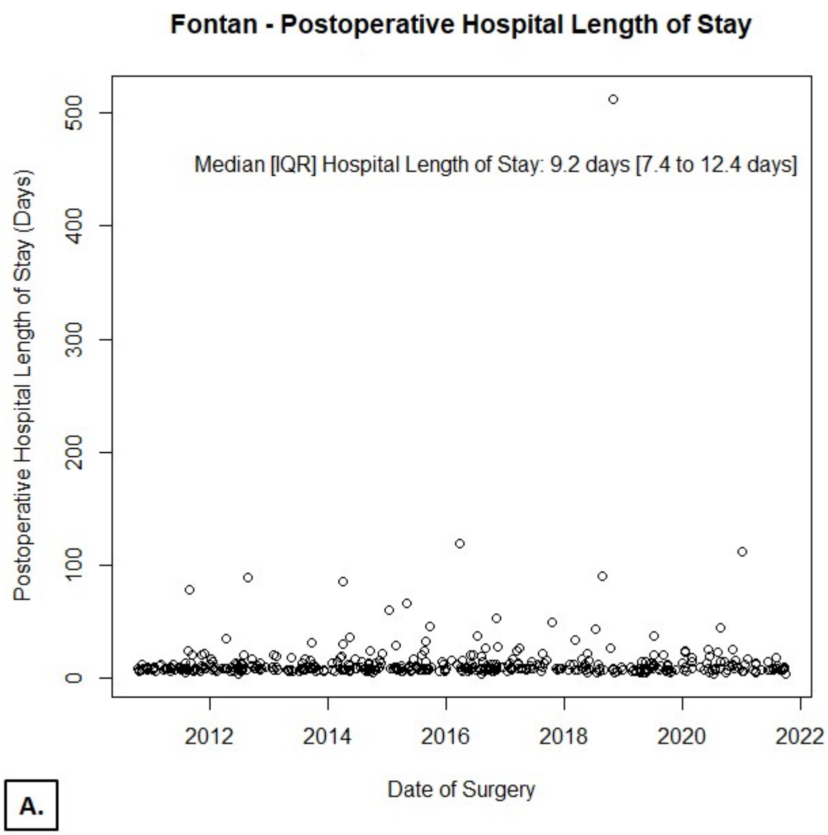

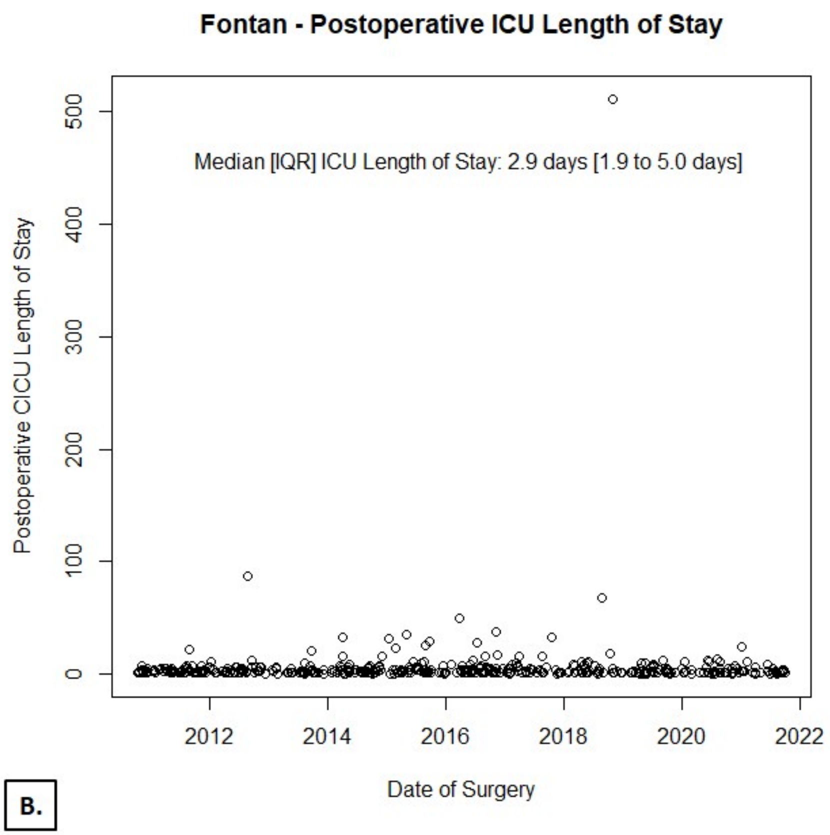

Supplemental Figure 5. Sensitivity analysis demonstrating that there was no decline in total postoperative hospital length of stay or ICU length of stay over time for patients undergoing the Fontan operation.

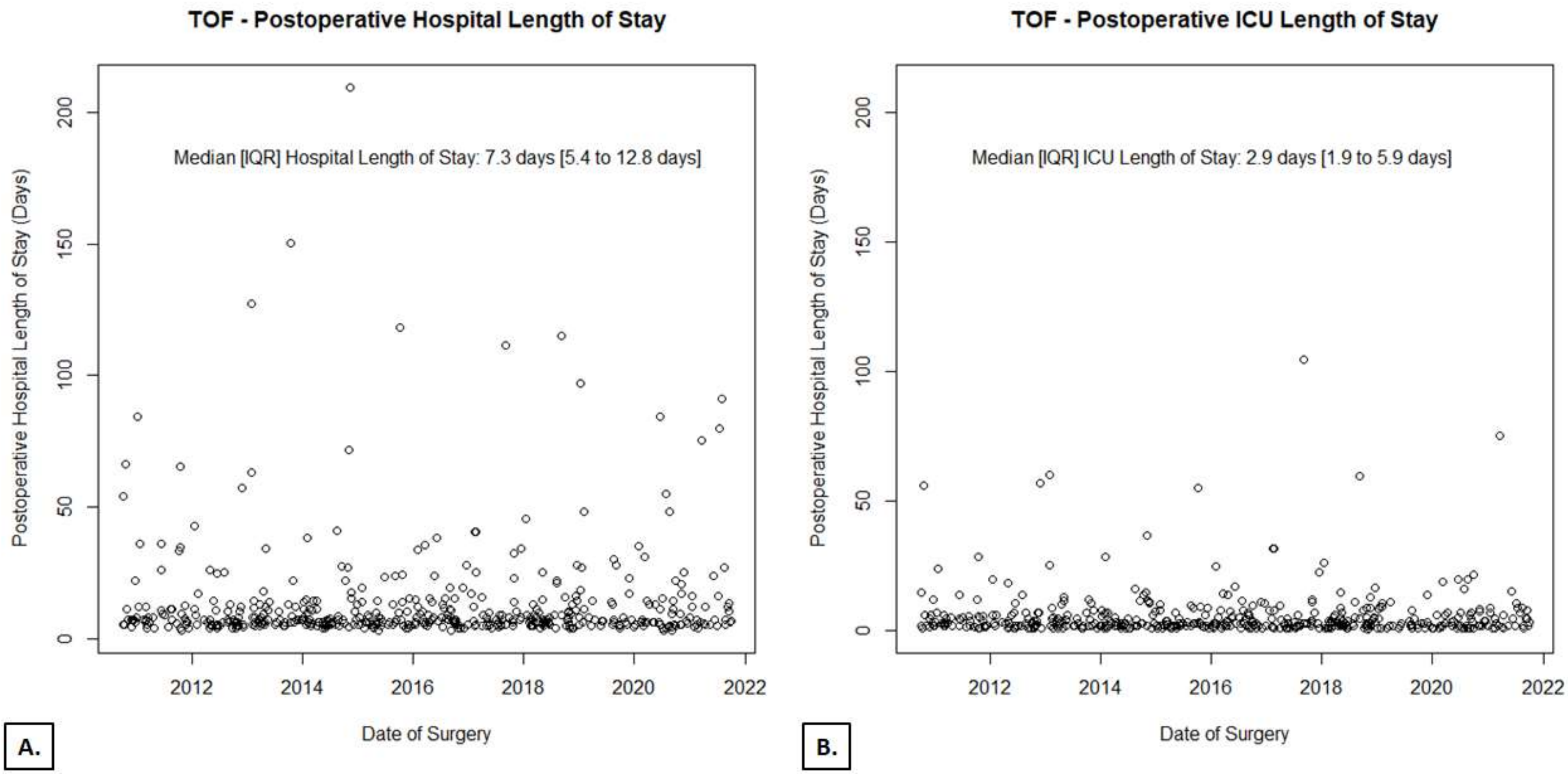

Supplemental Figure 6. Sensitivity analysis demonstrating that there was no decline in total postoperative hospital length of stay or ICU length of stay over time for patients undergoing the repair of Tetralogy of Fallot.

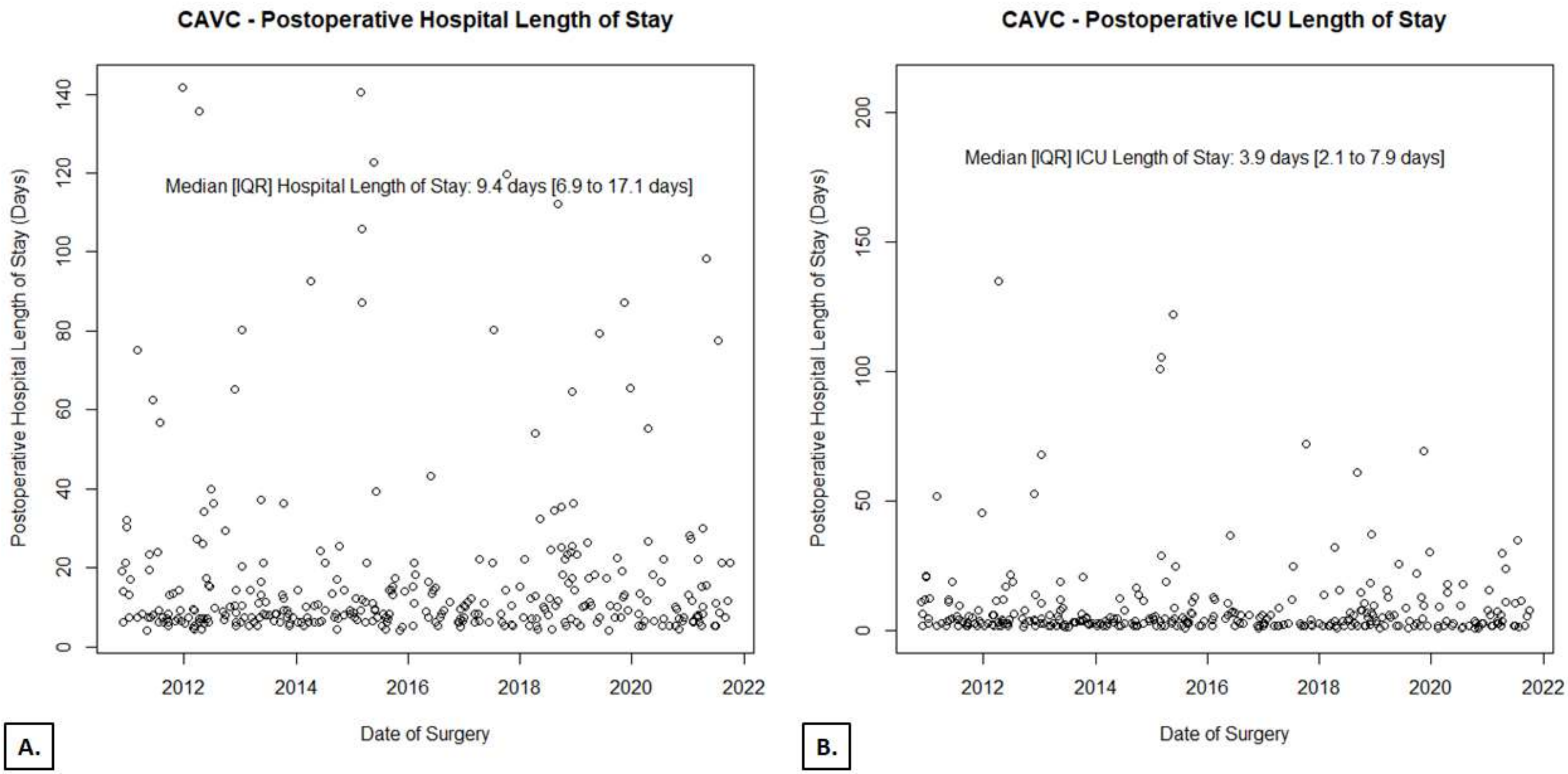

Supplemental Figure 7. Sensitivity analysis demonstrating that there was no decline in total postoperative hospital length of stay or ICU length of stay over time for patients undergoing the repair of complete atrioventricular canal.

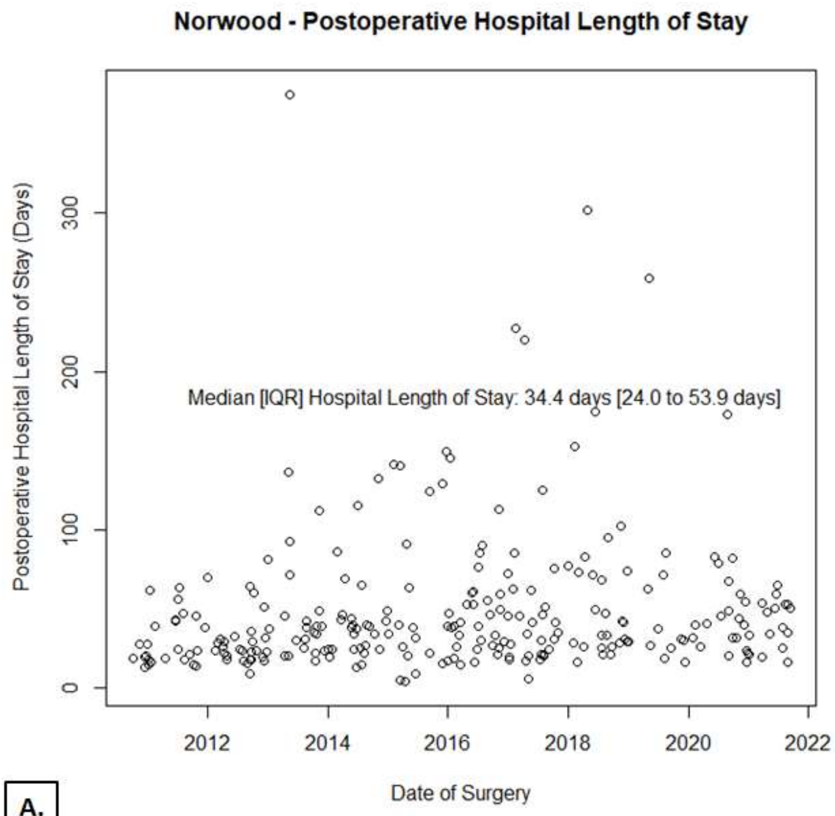

A.

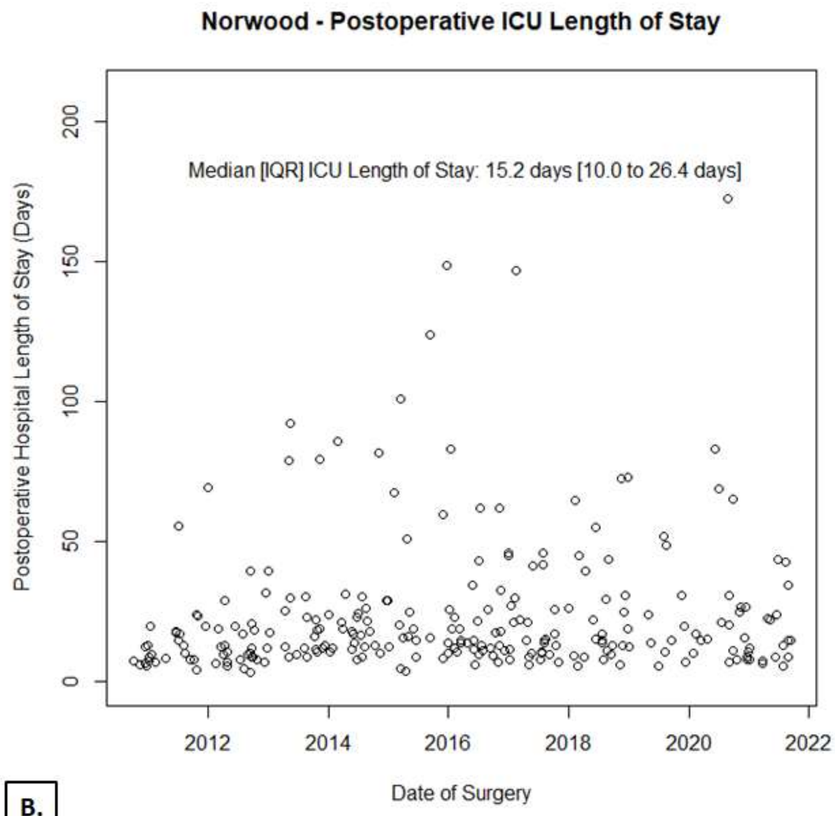

B.

Supplemental Figure 8. Sensitivity analysis demonstrating that there was no decline in total postoperative hospital length of stay or ICU length of stay over time for patients undergoing the Norwood operation.

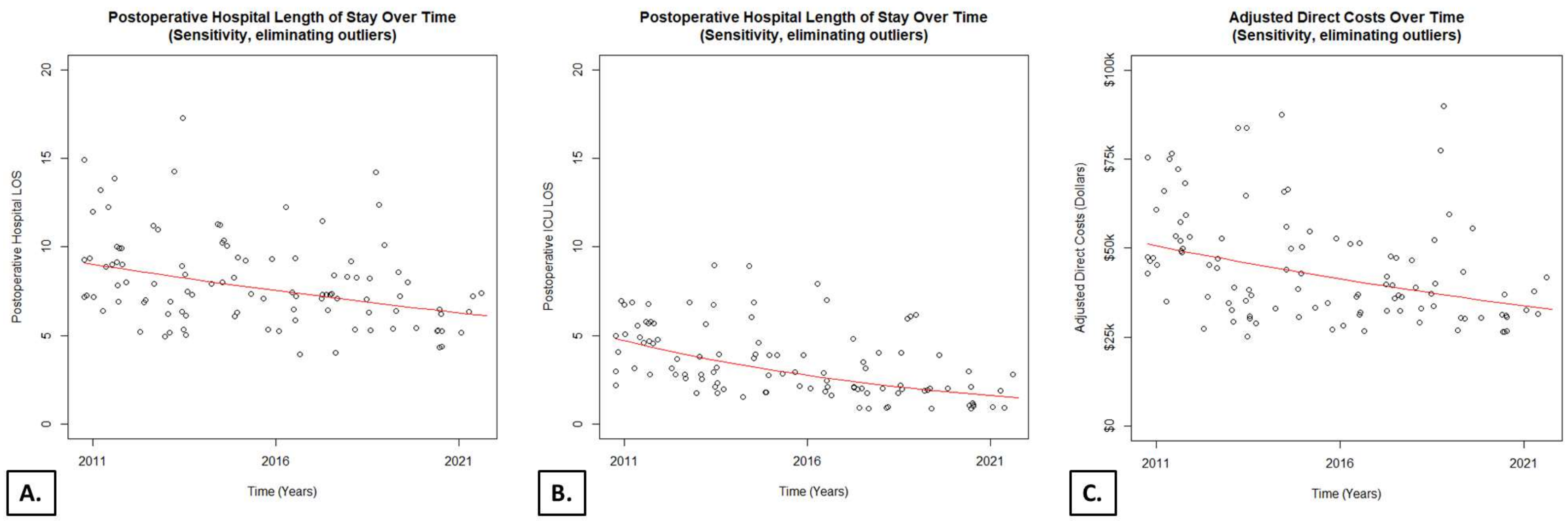

Supplemental Figure 9. In a sensitivity analysis after excluding cost outliers (patients in the 90th percentile and above), the exponential relationship was preserved.

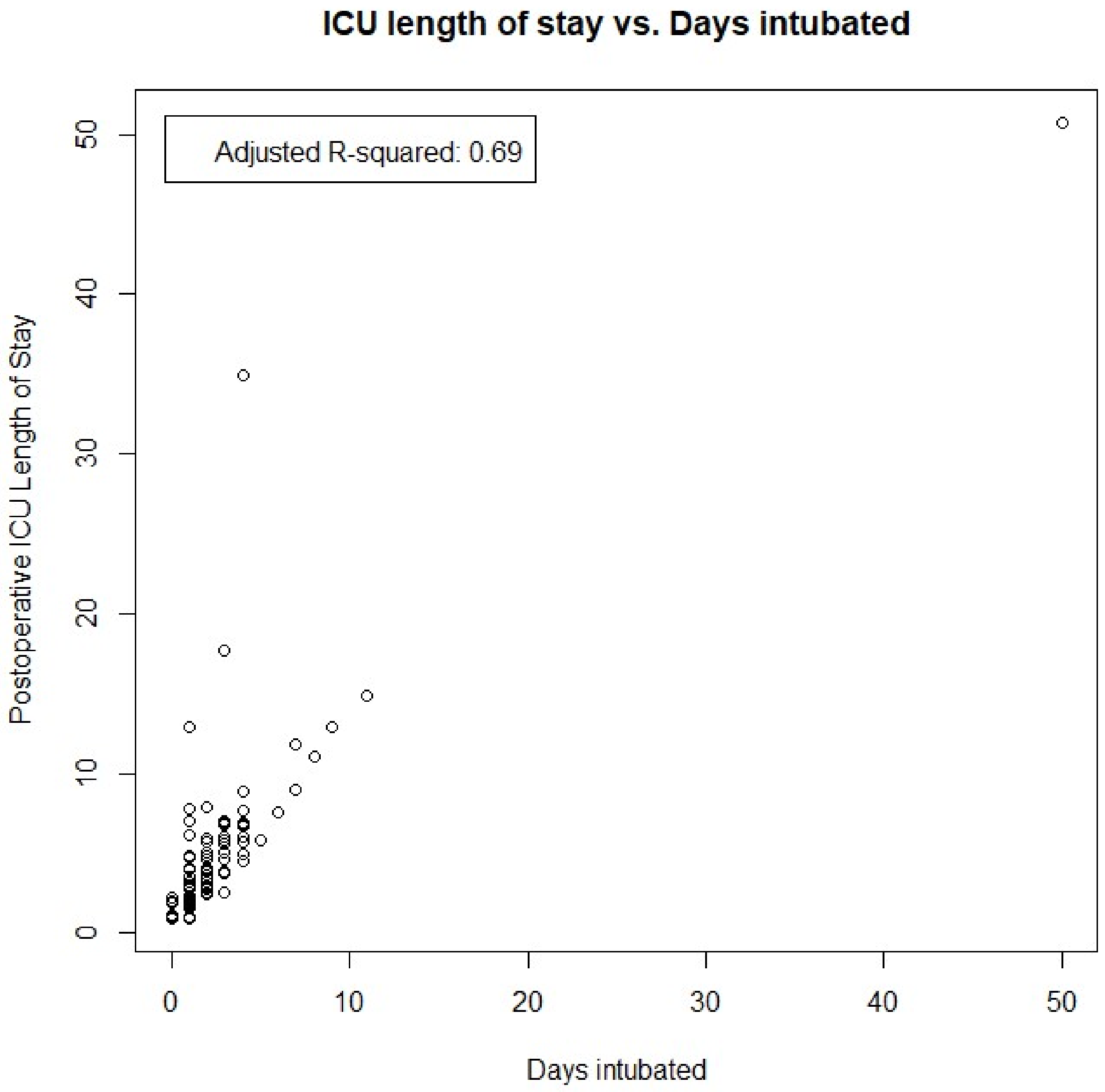

Supplemental Figure 10. Duration of ICU stay was correlated with the number of days intubated.
